# Label-Free Immunosensor Based on Liquid Crystal and Gold Nanoparticles for Cardiac Troponin I Detection

**DOI:** 10.3390/bios12121113

**Published:** 2022-12-02

**Authors:** Eduardo Zapp, Daniela Brondani, Tânia Regina Silva, Edivandro Girotto, Hugo Gallardo, Iolanda Cruz Vieira

**Affiliations:** 1 Department of Exact Science and Education, Federal University of Santa Catarina, Campus Blumenau, Blumenau 89036-256, Brazil; 2 Department of Chemistry, Federal University of Santa Catarina, Florianópolis 88040-900, Brazil

**Keywords:** troponin I, label-free immunosensor, columnar liquid crystal, gold nanoparticles

## Abstract

According to the World Health Organization (WHO), cardiovascular diseases (CVDs) are the leading cause of mortality and morbidity worldwide. The development of electrochemical biosensors for CVD markers detection, such as cardiac troponin I (cTnI), becomes an important diagnostic strategy. Thus, a glassy carbon electrode (GCE) was modified with columnar liquid crystal (LC_col_) and gold nanoparticles stabilized in polyallylamine hydrochloride (AuNPs–PAH), and the surface was employed to evaluate the interaction of the cTnI antibody (anti-cTnI) and cTnI for detection in blood plasma. Morphological and electrochemical investigations were used in the characterization and optimization of the materials used in the construction of the immunosensor. The specific interaction of cTnI with the surface of the immunosensor containing anti-cTnI was monitored indirectly using a redox probe. The formation of the immunocomplex caused the suppression of the analytical signal, which was observed due to the insulating characteristics of the protein. The cTnI–immunosensor interaction showed linear responses from 0.01 to 0.3 ng mL^−1^ and a low limit of detection (LOD) of 0.005 ng mL^−1^ for linear sweep voltammetry (LSV) and 0.01 ng mL^−1^ for electrochemical impedance spectroscopy (EIS), showing good diagnostic capacity for point-of-care applications.

## 1. Introduction

According to the World Health Organization (WHO), cardiovascular disease is the number one cause of death globally. Among cardiovascular diseases (CVDs), acute myocardial infarction (AMI), known as heart attack, is one of the main causes of morbidity and mortality worldwide. Early diagnosis of AMI is extremely important for a successful medical treatment of the condition and the prevention of fatality [1,2,3,4]. Today, cardiac troponins I and T are considered as the ‘gold standard’ biomarker for the detection of cardiac injury in the serological diagnosis and prognosis of AMI due to its remarkable sensitivity and specificity compared to other biochemical markers such as creatin kinase-MB isoenzyme and myoglobin [5,6]. Cardiac troponin I (cTnI) has the important advantage of being considered cardio-specific because it is exclusively expressed in the myocardial muscle. cTnI is released into the blood in a short time following muscle damage and persists in the blood for several days, providing an extended diagnostic window for the detection of AMI [7].

Conventional methods for monitoring the cTnI levels are widely used for AMI diagnosis, especially the typical enzyme-linked immunosorbent assay (ELISA) [8]. Currently, several other detection methods have been investigated for troponin quantification with high selectivity and sensitivity, among which surface-enhanced Raman spectroscopy (SERS) [9], surface plasmon resonance (SPR) [10], fluorescence [11], electrochemiluminescence [12], liquid chromatography/mass spectrometry (LC/MS) [13], and matrix-assisted laser desorption/ionization time-of-flight mass spectrometry (MALDI-TOF MS) [14]. However, these methods may have disadvantages, such as the long time required for analysis, multistep processing, high cost, and the requirement of sophisticated laboratory instrumentation [5,15]. Thus, in recent decades, researchers have sought methods to make said methods more practical, lowering cost, shortening analysis time, and achieving greater ease of miniaturization with high sensitivity and selectivity to enable a fast and safe diagnosis. Among the several techniques that have been investigated, electrochemical immunosensors have stood out as an important tool for the real-time monitoring of cardiac biomarkers [3,5,6,16,17,18,19,20]. Electrochemical methods, especially voltammetry and electrochemical impedance spectroscopy (EIS), are powerful techniques to monitor biorecognition events such as antigen-antibody complex formation, enabling a high sensitivity in the determination of biomarkers in real samples [16,20,21,22].

In recent years, significant improvements have been achieved in the performance of immunosensors with the use of nanomaterials [5,23,24,25], such as metal nanomaterials [26,27,28], quantum dots [16,20,29], carbon nanomaterials [3,30], and hybrid nanostructures [31]. The favorable electrical, chemical, and transport properties of these nanostructures have made them important materials for applications in sensor platforms, acting in the immobilization of antigens or antibodies and in signal amplification, enabling a more sensitive and selective electrochemical detection of biomarkers [23,24,25].

Liquid crystals (LCs) make up another class of materials widely exploited in various technological and research fields, including the development of biosensors [32,33,34]. The unique electro-optical properties of liquid crystal are promising toward the development of a simple and reliable sensing platforms for the quantification of biomolecules. For the most part, the optical properties of LC are exploited to detect molecules [33,35,36,37,38] and in a number of biosensing systems that employ electrochemical detection [39,40,41].

In the present study, a columnar LC (LC_col_) derived from 1,3,4-oxadiazole was applied in the development of an electrochemical immunosensor for the detection of cTnI. The immunosensor was constructed on a glassy carbon electrode (GCE), which received a first film of LC_col_ and a second film of gold nanoparticles stabilized in polyallylamine hydrochloride (AuNPs–PAH). This platform was used for covalent immobilization of anti-cTnI antibodies (activated with EDC/NHS), followed by blocking the nonspecific sites using glycine. Immunocomplex formation studies were performed using the Fe(CN)_6_^3−/4−^ redox probe as well as the voltammetric signal inhibition and measured by linear sweep voltammetry (LSV) or increased resistance to charge transfer (R_ct_), evaluated by EIS. After optimization, this immunosensor was applied to the determination of cTnI in samples of simulated serum and fortified blood plasma.

## 2. Materials and Methods

### 2.1. Reagents and Solutions

All reagents were of analytical grade and used as received without further purification. Cardiac troponin I antigen (cTnI) and cardiac troponin I antibody (ab-cTnI), chloroauric acid (HAuCl_4_), N-(3-dimethylaminopropyl)-N-ethylcarbodiimide hydrochloride (EDC), N-hydroxysuccinimide (NHS), poly(allylamine hydrochloride (PAH), potassium hexacyanoferrate (III) (K_3_Fe(CN)_6_), potassium hexacyanoferrate (II), (K_4_Fe(CN)_6_), and glycine (gly) were purchased from Sigma-Aldrich (São Paulo, Brazil). Ultrapure water was obtained from a Milli-Q system (Millipore, Bedford, MA, USA) at a resistivity of 18.2 MΩ cm^−1^ and used in the preparation of all solutions. Phosphate buffer saline (PBS) (0.01 mol L^−1^) was used as support electrolyte in the experiments, prepared by appropriate mixing of potassium chloride, sodium chloride, potassium phosphate monobasic, and sodium phosphate dibasic in 1000 mL of ultrapure water, with the pH value being adjusted with phosphoric acid or sodium hydroxide. Samples of lyophilized human blood plasma (Sigma-Aldrich) were spiked with antigen and used for the determination of cTnI using the electrochemical immunosensor.

For the study of possible interferents, the following solutions were prepared: creatinine (4.2 mg L^−1^), creatine (10.7 mg L^−1^), glucose (200 mg L^−1^), uric acid (10 mg L^−1^), ascorbic acid (10 mg L^−1^), bovine serum albumin (40 mg L^−1^), and myoglobin (10 ng mL^−1^), all purchased from Sigma-Aldrich.

**Figure 1:**
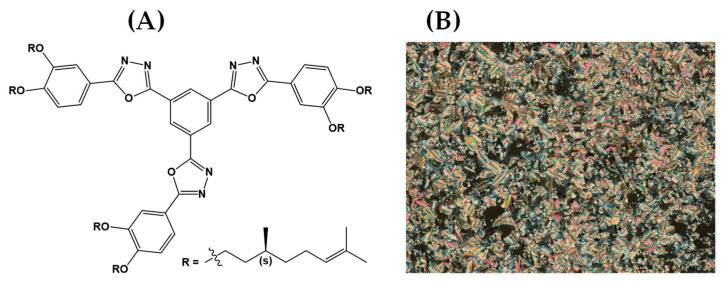
(**A**) The chemical structure of the LC_col_. (**B**) The optical photomicrograph (33×) of compound showing mosaic textural pattern for LC_col_ at 25 °C. Samples were sandwiched between untreated glass slides and viewed through crossed polarizers.

The AuNPs–PAH were synthesized as described by Silva and collaborators [42]. The obtained particles were characterized by transmission electronic microscopy (TEM). The aqueous sample of AuNPs–PAH was dispersed in an ultrasonic bath for 5 min and dropped onto a copper/carbon grid (300 mesh) placed on a sheet of paper; the sample was then air-dried and taken to the equipment for analysis. The LC_col_ molecules (Figure 1) were synthesized and characterized according to the methodology described by Girotto and collaborators [43].

### 2.2. Apparatus

All electrochemical measurements, namely cyclic voltammetry (CV), LSV, and EIS, were performed on a potentiostat/galvanostat model Autolab PGSTAT128N (Metrohm, Utrecht, The Netherlands), operating with a data-processing software (NOVA, software version 1.10). All voltammetry experiments were carried out using a conventional three-electrode system: the proposed immunosensor as working electrode, a platinum plate as the auxiliary electrode, and Ag/AgCl (3.0 mol L^−1^ KCl) as the reference electrode. The AuNPs–PAH samples were characterized by the transmission of electronic microscopy (TEM) (JEOL JEM-1011, Tokyo, Japan) at an acceleration voltage of 100 kV. The micrographs were obtained at the Central Laboratory of Electron Microscopy at the Federal University of Santa Catarina (Florianópolis, Brazil). Polarized optical microscopy (POM), used to characterize the sensor surface, was performed using a Leica DM4500 P (Wetzlar, Germany) optical microscope (using the IM50 software for data acquisition).

### 2.3. Construction of the Immunosensor

A GCE (2.0 mm in diameter) was polished successively with aqueous slurries of alumina (0.05 µm) on a flat pad for 2 min and rinsed with ultrapure water and ethanol. The GCE was then ultrasonically cleaned in ultrapure water for 5 min to remove the particles adsorbed over the electrode surface. Then, the electrode was washed with Milli-Q water and dried in a nitrogen stream to obtain a clean surface. Initially, activation of the ab-cTnI antibody with EDC and NHS was performed. In a microtube, solutions of EDC (5 × 10^−3^ mol L^−1^), NHS (2 × 10^−3^ mol L^−1^), and ab-cTnI (1000 ng mL^−1^) were mixed in a 1:1:1 ratio (*v*/*v*/*v*), and the solution was kept standing at 4 °C for 45 min. At the same time, the immunosensor assembly process was started. On the surface of a previously cleaned GCE (as described above), 3 μL of a solution of LC_col_ (1 mg mL^−1^ in dichloromethane) was dripped, and the solvent was allowed to evaporate into air at room temperature. After the formation of the LC_col_ film, the surface of the electrode was thermally treated in an oven (40 °C, 15 min) and then kept at room temperature for cooling and organization of the film for 15 min. In a second step, a suspension of AuNPs–PAH was dripped onto the surface of the electrode, and the solvent was evaporated under vacuum. Then, a 3 μL aliquot of the EDC/NHS pre-activated ab-cTnI solution was dripped onto the electrode surface. After 30 min, the electrode surface was washed with PBS to remove non-immobilized antibody molecules. Subsequently, the immunosensor was then incubated with 3 μL of gly (0.1 mol L^−1^) for 15 min to block non-active sites on the electrode surface.

### 2.4. Immunoassay Procedure

For studies with the cTnI immunosensor, human blood plasma samples (obtained from Sigma-Aldrich) were prepared with the addition of 0.05 or 0.1 ng mL^−1^ of cTnI. The performance of the developed cTnI immunosensor was analyzed at room temperature using LSV (based on current suppression) and EIS (based on R_ct_ magnitude) as a function of cTnI concentration in the sample analyzed. For the immunoassay, the reference signal was obtained (absence of the antigen). Subsequently, the immunosensor was incubated for 10 min with 3 μL of the sample containing cTnI. After the incubation step, the surface of the immunosensor was thoroughly washed with PBS (0.01 mol L^−1^, pH 7.5), and then a second signal was obtained by LSV or EIS.

## 3. Results

### 3.1. Fabrication and Sensing Principle of the Immunosensor

**Figure 2:**
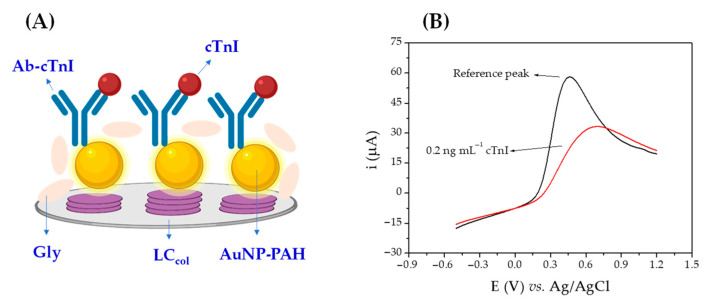
(**A**) Schematic representation of the immunosensor surface. (**B**) Voltammetric peaks in the absence and presence of cTnI.

The sensing principle for the detection of cTnI using the proposed immunosensor (Figure 2) is based on the inhibition of the voltammetric peak from the redox probe Fe(CN)_6_^3−/4−^ present in the analysis solution, when the molecules of cTnI adhere on the surface of the immunosensor through specific binding, forming an immunocomplex with the antibodies covalently immobilized on the nanostructured film (Figure 2A). This formation of the immunocomplex is responsible for the partial blocking of the electroactive surface, causing a greater electrical isolation of the layer on the electrode due to the poor conductive properties of the protein molecules (Figure 2B) [44]. Immunocomplex formation was monitored simultaneously by LSV, CV (faradaic electrochemical signal), and EIS (non-faradaic electrochemical signal). First, a linear voltammogram is obtained using the immunosensor in a PBS solution (0.01 mol L^−1^, pH 7.5) containing 1.0 × 10^−2^ mol L^−1^ of Fe(CN)_6_^3−/4−^ to obtain the base voltammetric peak (in the absence of cTnI). Subsequently, the immunosensor was incubated for 10 min with 3 µL of the cTnI-containing sample. After each step of the immunoassay, the surface of the immunosensor was thoroughly washed with PBS (0.01 mol L^−1^, pH 7.5) to remove any possible molecules that had been weakly and reversibly adsorbed, after which a second voltammetric peak was obtained. The peak inhibition percentage was correlated with the concentration of cTnI in the analyzed sample. The same experiment was simultaneously performed with EIS, wherein a base spectrum was initially obtained, and after the incubations with the samples containing cTnI, a second spectrum was obtained, the variation in R_ct_ being correlated with the amount of cTnI in the sample. The use of LC_col_ promotes an amplification of the measured impedimetric signal, making it possible to increase the sensitivity of the method [45].

### 3.2. Morphological Characterization

Supplementary Materials Figure S1 shows the TEM image of the as-prepared AuNPs–PAH. The obtained nanoparticles presented are roughly spherical, well dispersed in the grid, and show no sign of aggregation. The average diameter of the AuNPs–PAH was estimated to be approximately 17.8 ± 0.3 nm (using the measure of 900 particles arbitrarily chosen in the micrographs). As shown in Figure S1, the particle size distribution q was well fitted by a Gaussian curve.

**Figure 3:**
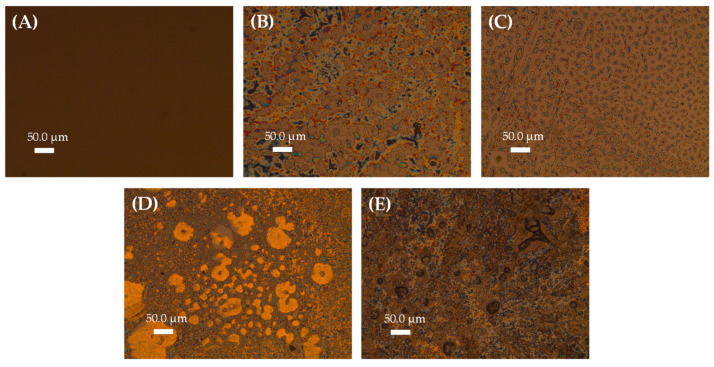
POM images for: (**A**) GCE, (**B**) LC_col_/GCE without heating, (**C**) LC_col_/GCE after heating to 40 °C, (**D**) AuNPs–PAH/LC_col_/GCE, and (**E**) ab-cTnI/AuNPs–PAH/LC_col_/GCE.

The surface of the proposed immunosensor was also analyzed by POM. Figure 3 shows the POM micrographs obtained in the different steps of immunosensor construction. In Figure 3A, we can clearly visualize the homogeneous clean surface of a bare GCE, while Figure 3B,C shows the effect of heat treatment (40 °C) on the organization of the LC_col_ film. In Figure 3D, after the formation of the AuNPs–PAH film, it is possible to observe the formation of small agglomerates of micrometer size for the film in solid form. Lastly, in Figure 3E, a change in the texture of the film is observed, indicating the presence of ab-cTnI molecules immobilized on the AuNPs–PAH film.

### 3.3. Electrochemical Studies

CV and EIS studies were carried out simultaneously to characterize the surface of the GCE modified with LC_col_, AuNPs–PAH, and ab-cTnI activated with EDC/NHS. No redox peak was observed for the LC_col_/GCE in PBS (0.01 mol L^−1^, pH 7.5) (data not shown); therefore, a redox probe in solution (Fe(CN)_6_^3−/4−^) was used. Figure S2A shows the CV curves for the (a) bare GCE, (b) LC_col_/GCE without heating, and (c) LC_col_/GCE after heating to 40 °C in Fe(CN)_6_^3−/4−^ 1.0 × 10^−2^ mol L^−1^ prepared in PBS (0.01 mol L^−1^, pH 7.5). Figure S2A shows the effect of the heat treatment at 40 °C for the LC_col_/GCE, since the LC_col_ layer on GCE (without heating) (voltammogram b) causes a small suppression of the CV signal of the redox probe of about 9% when compared to the bare GCE. After the heat treatment of the LC_col_/GCE (voltammogram c), it was possible to again observe a reduction in the peak current of about 15% in relation to the bare GCE (voltammogram a). This modification was also accompanied by an increase in peak potentials. The same can be assessed by EIS (Figure S2B), which shows an increase in R_ct_ when the same treatment was performed.

**Figure 4:**
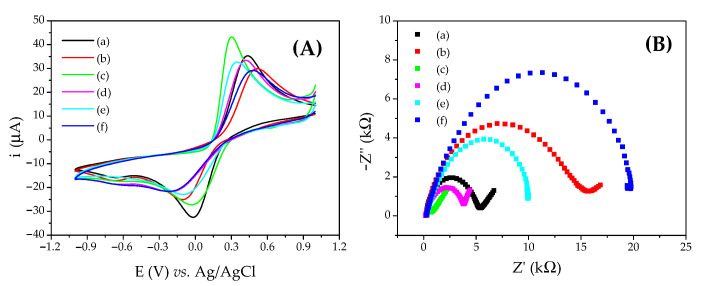
(**A**) Cyclic voltammograms obtained with a scan rate of 100 mV s^−1^ and (**B**) Nyquist diagrams obtained in open circuit mode, with 10 mV amplitude and frequency range from 0.1 to 100,000 Hz for: (a) bare GCE; (b) LC_col_/GCE after heating to 40 °C; (c) AuNPs–PAH/LC_col_/GCE; (d) ab-cTnI/AuNPs–PAH/LC_col_/GCE; (e) gly/ab-cTnI/AuNPs–PAH/LC_col_/GCE; and (f) 0.3 ng mL^−1^ cTnI incubated on gly/ab-TnI/AuNPs–PAH/LC_col_/GCE in PBS (0.01 mol L^−1^, pH 7.5) containing 1.0 × 10^−2^ mol L^−1^ of Fe(CN)_6_^3−/4−^.

The CV measurements of the electroactive species such as Fe(CN)_6_^3−/4−^ are a valuable and convenient tool to monitor the blocking effect of the surface of the modified electrode, since the transfer of electrons between the electrode and the species in solution is dependent on the degree of coverage of the conductive surface [46]. The degree of inhibition of the faradaic process is closely related to the electrochemical properties of the films deposited on the electrode surface. This behavior was investigated by CV and EIS. Figure 4A shows the cyclic voltammograms after each assembly step on the LC_col_/GCE platform. Figure 4A shows the formation of the LC_col_ film on GCE surface (voltammogram b), promoting a reduction in the anodic (i_pa_) and cathodic (i_pc_) peak currents for Fe(CN)_6_^3−/4−^ in relation to the bare GCE (voltammogram a). For the anodic peak, the drop was of 15% after heat treatment. The addition of AuNPs–PAH to the LC_col_/GCE (Figure 4A—voltammogram c) causes a 23% increase in the voltammetric response due to the higher conductivity of the nanostructures. After immobilization of the ab-cTnI antibody to the electrode modified with AuNPs–PAH/LC_col_/GCE, a reduction in currents of about 22% and an increase in peak separation for the redox probe Fe(CN)_6_^3−/4−^ in PBS (0.01 mol L^−1^, pH 7.5) was observed (Figure 4A—voltammogram d) due to the insulating characteristics of the protein [44]. The adsorption of gly (Figure 4A—voltammogram e) to block non-specific binding sites promoted a current reduction of 5.5% in relation to electrode d. After the incubation of the gly/ab-cTnI/AuNPs–PAH/LC_col_/GCE with 0.3 ng mL^−1^ of cTnI (Figure 4A—voltammogram f), a further reduction in the voltammetric peaks of around 10% was observed due to the formation of the immunocomplex on the immunosensor surface. The same process can be better observed by EIS (Figure 4B).

### 3.4. Immunosensor Analytical Performance

In order to obtain the best operating conditions for the proposed immunosensor, some parameters were investigated using the redox probe Fe(CN)_6_^3−/4−^ for the evaluation of experimental parameters. The parameters evaluated were as follows: selection of electrochemical technique; amount of LC_col_; amount of AuNPs–PAH; association constant; and incubation time.

#### 3.4.1. Selection of the Electrochemical Technique

In order to select the best technique for using the immunosensor, three different electrochemical techniques were evaluated: LSV, SWV, and EIS (data not shown). The selection criteria were obtaining an expressive signal for use with a faradaic inhibition process and, mainly, the reproducibility of the responses obtained for each technique in relation to the baseline peak signal (in the absence of cTnI), thus avoiding a false result when the immunosensor is incubated with the cTnI antigen. For this, three successive experiments were carried out with each technique in PBS (0.01 mol L^−1^, pH 7.5) containing 1.0 × 10^−2^ mol L^−1^ of Fe(CN)_6_^3−/4−^. The results obtained with LSV showed a constant signal behavior with little variation in the current signal between the three measurements performed. The same behavior was observed for EIS. For the SWV study, the signal obtained for the same system was not stable, which makes its use for detection by the immunosensor unfeasible. Based on these results, LSV and EIS were selected for use in detecting cTnI by the proposed immunosensor.

#### 3.4.2. Platform Redox Response Optimization

The dependence on the amount of LC_col_ for the immunosensor response was evaluated using LC_col_ solutions with different concentrations (0.1 to 10 mg mL^−1^) (Figure S3A). The best response was obtained using a solution with a concentration equal to 1.0 mg mL^−1^ and above 2 mg mL^−1^. A 100% suppression of the voltammetric signal from the Fe(CN)_6_^3−/4−^ probe was observed.

The effect of AuNPs–PAH on the sensor response was evaluated based on different proportions of AuNPs–PAH. For this study, the dispersion of AuNPs–PAH was concentrated in the following ratios: 10×, 5×, 2× and 0×. Figure S3B shows the results of the investigated AuNPs–PAH proportions, with the relative response measured as a function of a 1.0 × 10^−2^ mol L^−1^ solution of Fe(CN)_6_^3−/4−^ in PBS (0.01 mol L^−1^, pH 7.5). As shown, the peak cathodic current increases with increasing concentrations, reaching a maximum level for a factor of 5×. For higher dilution values, the response to the redox probe shows a decrease. Thus, the proportion of AuNPs–PAH with a factor of 5× was chosen for the other studies.

#### 3.4.3. Incubation Time and Association Constant

For this study, a range of 2 to 60 min incubating the immunosensor with 0.3 ng mL^−1^ of cTnI was used. Incubation time was evaluated as the time required to generate a significant percentage inhibition of redox probe response in a region before the complete suppression of the signal (Figure S4A). The incubation time selected was 10 min, as the suppression of the generated signal is already sufficient to follow the events of immunocomplex formation.

The apparent association constant (k_ass_^app^) for the formation of the immunocomplex was obtained from the data in Figure S4B,C, and using the equation described by Szymańska and collaborators [47]. The relationship between the occupied binding sites (*θ*) and the change in R_ct_ is given by Equation (1) [48].

(1) $$\theta =1-\frac{R_{\mathrm{ct}}^{\left(0\right)}}{R_{\mathrm{ct}}^{\left(i\right)}}$$

where R_ct_^(0)^ and R_ct_^(i)^ are the resistance to charge transfer in the absence (reference spectrum) and in the presence of the antigen, respectively. In the case of Langmuir isotherms, the *θ* values can be related to the association constant according to Equation (2):

(2) $$\theta =\frac{K_{\mathrm{ass}}\times \left[\mathrm{cTnI}\right]}{1-K_{\mathrm{ass}}\times \left[\mathrm{cTnI}\right]}$$

where k_ass_ is the association constant and [cTnI] is the concentration of the target molecule. The linearization of the Langmuir isotherm provides Equation (3):

(3) $$K_{\mathrm{ass}}\times \left[\mathrm{cTnI}\right]=\frac{\theta }{1-\theta }$$

Combining Equations (1) and (3), we obtain Equation (4):

(4) $$K_{\mathrm{ass}}\times \left[\mathrm{cTnI}\right]=\frac{R_{\mathrm{ct}}^{\left(i\right)}-R_{\mathrm{ct}}^{\left(0\right)}}{R_{\mathrm{ct}}^{\left(0\right)}}$$

The value of R_ct_^(i)^ varies linearly with the concentration of cTnI from 4.18 × 10^−12^ to 1.26 × 10^−11^ mol L^−1^ (Figure S4C). Therefore, the association constant can be calculated from the slope of the curve of (R_ct_^(i)^ − R_ct_^(0)^)/R_ct_^(0)^ vs. [cTnI] (mol L^−1^). The k_ass_^app^ value found for the electrochemical measurement was 1.38 × 10^11^ L mol^−1^.

#### 3.4.4. Calibration Curve for cTnI

**Figure 5:**
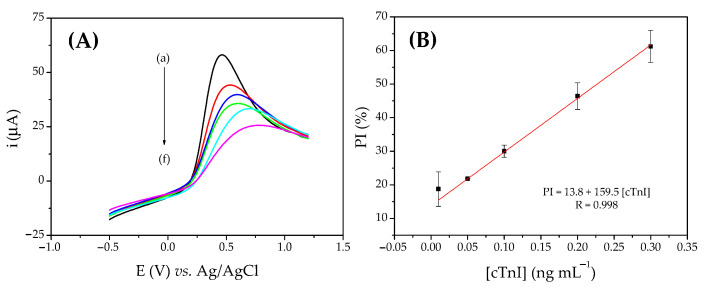
(**A**) Linear voltammograms obtained with a scan rate of 100 mV s^−1^ with the immunosensor for cTnI in (a) PBS (0.01 mol L^−1^, pH 7.5) containing 1.0 × 10^−2^ mol L^−1^ of Fe(CN)_6_^3−/4−^ and after 10 min of incubation with: (b) 0.01, (c) 0.05, (d) 0.1, (e) 0.2, and (f) 0.3 ng mL^−1^ cTnI. (**B**) Voltammetric calibration curve for cTnI.

**Figure 6:**
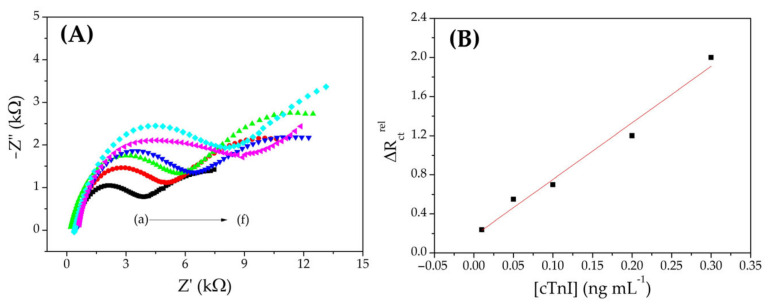
(**A**) Nyquist diagrams obtained in open circuit mode, with 10 mV amplitude and frequency range of 0.1–100,000 Hz with the immunosensor for cTnI in (a) PBS (0.01 mol L^−1^, pH 7.5) containing 1.0 × 10^−2^ mol L^−1^ of Fe(CN)_6_^3−/4−^ and after 10 min of incubation with: (b) 0.01, (c) 0.05, (d) 0.1, (e) 0.2, and (f) 0.3 ng mL^−1^ cTnI. (**B**) Impedimetric calibration curve for cTnI.

Under optimized conditions, calibration curves were obtained with increasing concentrations of cTnI between 0.01 and 0.3 ng mL^−1^ using LSV (Figure 5) and EIS (Figure 6). The linear regression equation obtained for the voltammetric curve was PI = 13.8 (±0.28) + 159.5 (±5.6) [cTnI] with a correlation coefficient of 0.998, where PI is the percentage of inhibition of the voltammetric signal (%) and [cTnI] is the concentration of cardiac troponin I (ng mL^−1^).

The impedimetric curve presented a linear regression equation of: ΔR_ct_^rel^ = 0.19 (± 0.05) + 5.34 (± 0.4) [cTnI], with a correlation coefficient of 0.990, where ΔR_ct_^rel^ is the variation of charge transfer resistance in relation to the reference spectrum (R_ct_^(i)^ − R_ct_^(0)^/R_ct_^(0)^) and [cTnI] is the cardiac troponin I concentration (ng mL^−1^). The calculated limits of detection (LOD) were 0.005 ng mL^−1^ and 0.01 ng mL^−1^ for LSV and EIS, respectively.

### 3.5. Reproducibility, Study of Possible Interfering Compounds, and Application of the Immunosensor

**Figure 7:**
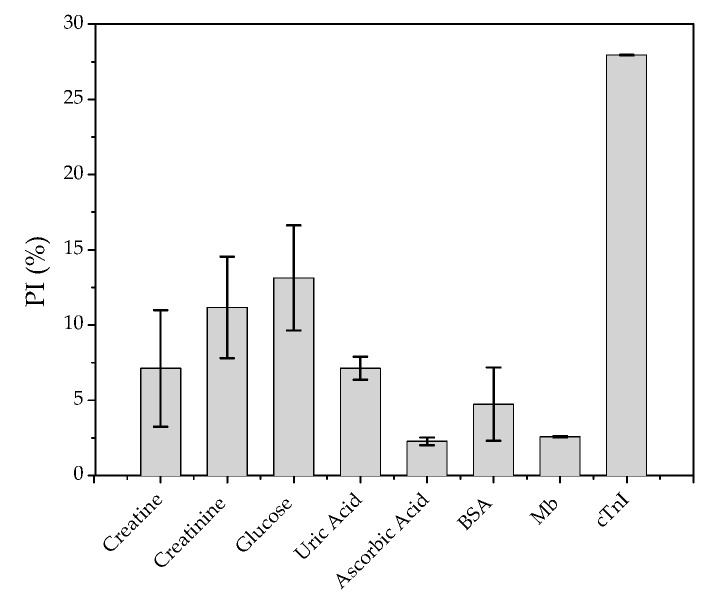
Study of potentially interfering compounds present in a biological sample.

The reproducibility of the proposed immunosensor was evaluated by detecting 0.05 ng mL^−1^ of cTnI, with the relative standard deviation for six individual measurements equal to 8.1%. In order to investigate possible interferences, a study was carried out with potentially interfering compounds that are found in biological samples (Figure 7).

The analytical response for the developed immunosensor was obtained by incubating the electrode in blood plasma samples containing cTnI. The results for the analyzed samples are shown in Table 1.

Table 2 presents the results obtained for other immunosensors for cTnI based on various methods and materials previously described in the literature. It can be seen from the table that the proposed immunosensor has a low LOD and short diagnostic time.

## 4. Discussion

The success of a biosensor used in diagnostic monitoring lies in its ability to detect even the smallest physiological change with precision, sensitivity, low analysis time, and low level of interference. In order to achieve these goals, it is essential to know the structure of the immunosensor construction, as well as its behavior. Thus, we started this process by evaluating the morphology of the materials used in the construction of the active layers of the developed immunosensor. Figure 3 clearly shows a structural change due to the heating effect to which the sensor was subjected. Considering that the LC_col_ used has a transition temperature to columnar mesophase close to 30 °C [43] and the sensor surfaces were treated with a temperature equal to 40 °C, the change observed in Figure S2 was considered as evidence of the organization of the LC_col_ on the GCE’s surface—this same structure being retained when the temperature is reduced to room temperature. Furthermore, the formation of the immobilization layer by AuNPs–PAH (Figure S1) formed small agglomerates that could be observed in the micrograph, proving that this layer adhered properly to the organized layer of LC_col_, in addition to the presence of the polyelectrolyte PAH allowing for bioreceptor immobilization. The presence of ab-cTnI molecules covalently immobilized using EDC/NHS to favor correct binding of antibodies can be observed by the change in texture observed in the micrographs.

All the construction steps were followed by electrochemical characterization, as shown in Figure 4. After the heat treatment of the LC_col_/GCE, it was possible to observe a reduction in the peak current to about 15% in relation to the bare GCE—evidence of a kinetically slower process. This modification was also accompanied by an increase in peak potentials—evidence that the process is becoming thermodynamically more unfavorable, which is attributed to the stacking of LC_col_ molecules in columns, generating an energy gap [32]. The same can be assessed regarding EIS (Figure S2B), which shows an increase in R_ct_ when the same treatment was performed. Although there is a small blockage of the electrode surface by the film, it plays an important role in the development of the immunosensor, as the use of this semiconductor material promotes an amplification of the resistance response, which is used for the detection of cTnI by EIS. In addition, the AuNPs–PAH layer, used for the immobilization of ab-cTnI antibodies, interacts with this LC_col_ layer through intermolecular interactions. The combination of LC_col_ and AuNPs–PAH proved to be more stable than the materials used individually with the ab-cTnI. The use of AuNPs–PAH, in addition to being used for the immobilization of ab-cTnI, also contributed to the improvement of the electrochemical signal, resulting in an increase in the voltammetric signal, evidencing a facilitation of the electronic transfer process promoted by the addition of metallic nanostructures.

Based on the best voltammetric response of the electrode, the construction and operating conditions were selected. With the optimization of the immunosensor, the best operating conditions could be obtained to increase the detection capacity of the immunosensor. For LC_col_, the criterion used for this selection was the voltammetric peak profile—an amount of LC_col_ was used that promotes a coating of the GCE surface, but not enough to electrically insulate the electrode. For AuNPs–PAH, a facilitating effect was observed in the electronic transfer promoted by the addition of metallic nanostructures; as such, the AuNPs quantity that caused the best increase in the voltammetric signal was selected. The incubation time is directly related to the diagnosis time and is therefore an important operational parameter to be evaluated. For the values evaluated, 10 min was the incubation time sufficient to cause a detectable response to the immunosensor. Furthermore, when compared to other previously published works (Table 2), this amount of time has shown to be relatively low, which favors a rapid diagnosis using cTnI detection.

Using EIS, the k_ass_^app^ value was measured, the value found being equal to 1.38 × 10^11^ L mol^−1^, which suggests that the formation of the immunocomplex is greatly favored, and therefore the immobilization method guaranteed a correct immobilization and orientation of the antibody on the electrode surface.

The process results from the specific biointeraction of the antibody with its antigen, considering that the protein causes a partial blockage of the surface, caused a reduction in the charge transfer on the electrode surface, and therefore an increase in R_ct_ and a decrease in the intensity of the voltammetric response—this being the principle of cTnI recognition by the immunosensor. This strategy was successfully used to obtain the calibration curves. As shown in Figure 5 and Figure 6, the two methods employed showed good linearity between immunosensor responses and different cTnI concentrations (0.01–0.3 ng mL^−1^), with adequate linearity. Furthermore, the LOD values obtained are comparable to the cut-off values of 0.03 ng mL^−1^ [52]. Therefore, the obtained working range and the LOD are sufficient to identify a possible IAM.

Reproducibility and specificity are important analytical parameters for a sensor. Under optimized conditions, a concentration of 0.05 ng mL^−1^ of cTnI was detected under six repetitions in individuals, which presented a relative standard deviation of 8.1%, verifying the accuracy of the immunosensor proposed. The specificity of the immunosensor is also a critical parameter of operation, for which different interferents were evaluated, as described above. As seen in Figure 7, only creatinine and glucose caused a noticeable decrease in peak signal, slightly greater than 10%. The other tested interferents showed a response of less than 10% when compared to the response with cTnI, including with another cardiac marker, Mb, showing that the studied immunosensor has greater selectivity for cTnI.

Then, spiked plasma samples were analyzed under optimized conditions. As shown in Table 1, the proposed immunosensor was able to detect with good accuracy the concentration of cTnI in plasma samples.

These data indicate that the immunosensor has potential for use in guiding diagnoses, serving as a complementary tool to other clinical methods.

## 5. Conclusions

The present work presented the development of a label-free electrochemical immunosensor containing LC_col_ and AuNPs–PAH, which was evaluated and applied in the detection of cTnI, an important cardiac biomarker used in the diagnosis of AMI. Based on the studies carried out, it was possible to evaluate the use of a molecule of LC_col_ with a transition to mesophase at a low temperature (30 °C), which proved to be efficient for the formation of a layer for immobilization, in addition to promoting the amplification of the impedimetric signal. It was also possible to observe the efficient immobilization of ab-cTnI antibodies with AuNPs stabilized in PAH using EDC/NHS as a binding reagent, evidenced by the high value of the association constant determined for the formation of the immunocomplex. The immunosensor was successfully optimized, and under optimized conditions it was able to detect cTnI in the range of 0.01 to 0.3 ng mL^−1^ with an LOD of 0.005 ng mL^−1^ (LSV) and 0.01 ng mL^−1^ (EIS). The proposed device was successfully used in the determination of cTnI in blood plasma samples with low interference and good reproducibility of the results, indicating that the developed immunosensor can detect acceptable clinical levels of cTnI with low analysis time, proving to be a tool useful for the diagnosis of AMI.

## Figures and Tables

**Table 1:** Determination of cTnI in blood plasma—data obtained from the voltammetric method.

Sample	[cTnI] Detected (ng mL^−1^) *	Relative Error (%)
Plasma (0.05 ng mL^−1^ of cTnI)	0.056 ± 0.007	12.0
Plasma (0.05 ng mL^−1^ of cTnI)	0.049 ± 0.002	−2.0
Plasma (0.1 ng mL^−1^ of cTnI)	0.095 ± 0.019	−5.0

* Mean ± standard deviation (for triplicate).

**Table 2:** Comparison of different immunosensors for cTnI.

Method *	Material	Linear Range (ng mL^−1^)	LOD (ng mL^−1^)	Incubation Time (min)	Reference
Electrochemical	WNFs/GCE	0.5–100	0.04	110	[3]
Electrochemical	APTES/WO_3_-RGO/ITO	0.01–250	0.01	10	[49]
Electrochemical	AuPtPd FNDs/GCE	0.01–100	0.003	80	[50]
WGM	LC	0–40	1.103	30	[51]
Electrochemical	AuNPs–PAH/LC_col_/GCE	0.01–0.3	0.005 (LSV)MILOS0.01 (EIS)	10	This work

* WNFs: carbon nanotube-whiskered nanofibers; GCE: glassy carbon electrode; WO3-RGO: tungsten trioxide-reduced graphene oxide; ITO: indium tin oxide; AuPtPd FNDs: AuPtPd porous fluffy-like nanodendrites; WGM: whispering gallery mode; LC: liquid crystal.

## Data Availability

Not applicable.

## Supplementary Materials

The following supporting information can be downloaded at: https://www.mdpi.com/article/10.3390/bios12121113/s1. Figure S1: (A) TEM micrograph of the AuNPs–PAH. (B) Particle size histogram based on approximately 900 particles; Figure S2: (A) Cyclic voltammograms obtained at scan rate of 100 mV s^−1^. (B) Nyquist diagrams obtained in open-circuit mode, with 10 mV amplitude and frequency range of 0.1–100,000 Hz for: (a) bare GCE, (b) LC_col_/GCE without heating, and (c) LC_col_/GCE with heating (40 °C) in PBS (0.01 mol L^−1^, pH 7.5) containing 1.0 × 10^−2^ mol L^−1^ of Fe(CN)_6_^3−/4−^; Figure S3: (A) Effect of the concentration of LC_col_ (0.1 to 10 mg mL^−1^) on the response of the sensor. (B) Effect of the amount of AuNPs–PAH in different proportions (0×, 2×, 5× and 10×). Measurements obtained in PBS (0.01 mol L^−1^, pH 7.5) containing 1.0 × 10^−2^ mol L^−1^ of Fe(CN)_6_^3−/4−^, with a scan rate of 100 mV s^−1^; Figure S4: (A) Percentage of inhibition as a function of time. (B) Nyquist diagram obtained in open-circuit mode, with 10 mV amplitude and frequency range of 0.1–100,000 Hz for gly/ab-cTnI/AuNPs–PAH/LC_col_/GCE for incubation with 0.3 ng mL^−1^ cTnI in PBS (0.01 mol L^−1^, pH 7.5) containing 1.0 × 10^−2^ mol L^−1^ of Fe(CN)_6_^3−/4−^ to evaluate the effect of incubation time (2 to 60 min). (C) Effect of cTnI concentration obtained in the range from 4.18 × 10^−12^ to 1.26 × 10^−11^ mol L^−1^ with incubation time of 10 min as a function of the variation in R_ct_.
