# Supplementary material for: Label-Free Immunosensor Based on Liquid Crystal and Gold Nanoparticles for Cardiac Troponin I Detection
Source: Biosensors (Basel). 2022 Dec 2;12(12):1113. doi: 10.3390/bios12121113 (PMC9775587; doi:10.3390/bios12121113)
Supplement: Supplementary file 1 [file biosensors-12-01113-s001.zip › biosensors-2022519-supplementary.pdf]

biosensors

Supporting Information

Label-Free Immunosensor Based on Liquid Crystal and Gold Nanoparticles for Cardiac Troponin I Detection

Eduardo Zapp [1], Daniela Brondani [1], Tânia Regina Silva [2], Edivandro Girotto [2], Hugo Gallardo [2] and Iolanda Cruz Vieira [2,*]

[1] Department of Exact Science and Education, Federal University of Santa Catarina, Campus Blumenau, Blumenau 89036-256, Brazil
[2] Department of Chemistry, Federal University of Santa Catarina, Florianópolis 88040-900, Brazil
* Correspondence: iolanda.vieira@ufsc.br

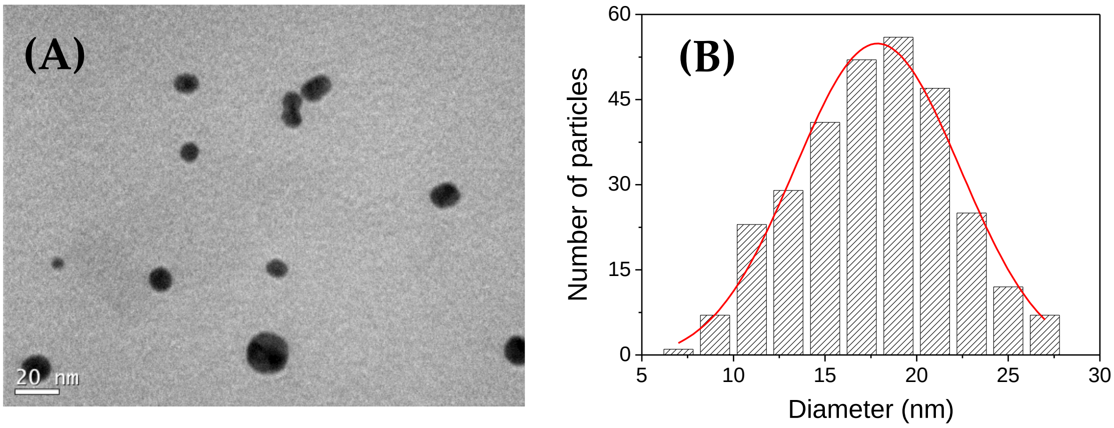

Fig. S1. (**A**) TEM micrograph of the AuNP-PAH. (**B**) Particle size histogram based on approximately 900 particles.

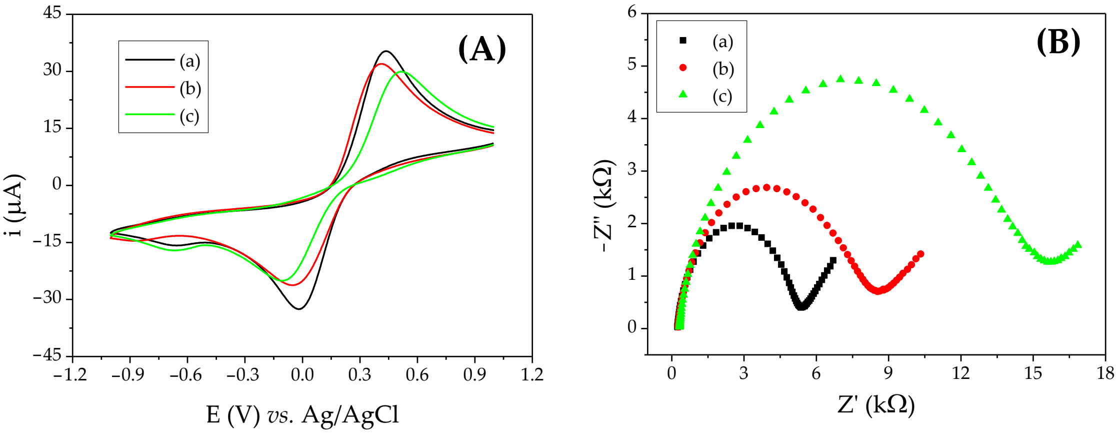

Figure S2. (**A**) Cyclic voltammograms obtained at scan rate of 100 mV s^{-1}. (**B**) Nyquist diagrams obtained in open-circuit mode, with 10 mV amplitude and frequency range of 0.1–100,000 Hz for: (a) bare GCE, (b) LC_{col}/GCE without heating and (c) LC_{col}/GCE with heating (40 °C) in PBS (0.01 mol L^{-1}, pH 7.5) containing 1.0 × 10^{-2} mol L^{-1} of $Fe(CN)_6^{3-/4-}$.

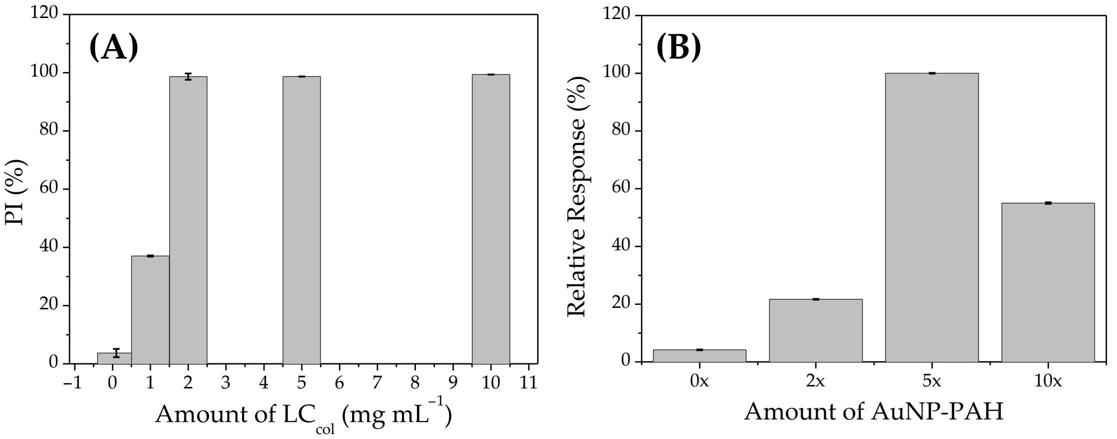

Figure S3. (**A**) Effect of the concentration of LC_{col} (0.1 to 10 mg mL^{-1}) on the response of the sensor. (**B**) Effect of the amount of AuNP-PAH in different proportions (0×, 2×, 5× and 10×). Measurements obtained in PBS (0.01 mol L^{-1}, pH 7.5) containing 1.0 ×10^{-2} mol L^{-1} of $Fe(CN)_6^{3-/4-}$, with a scan rate of 100 mV s^{-1}.

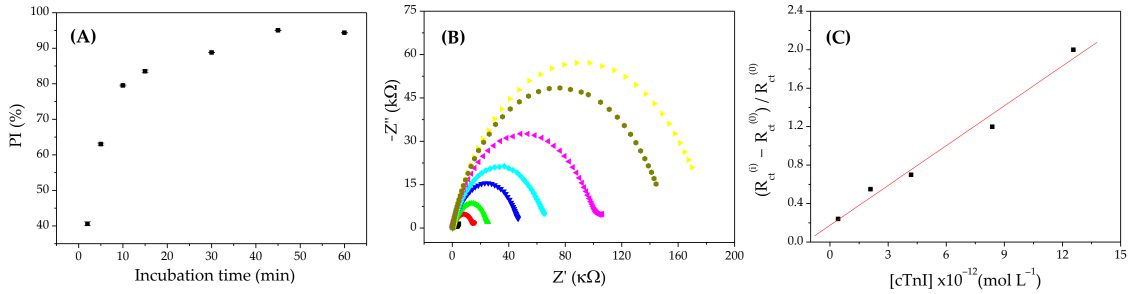

Figure S4. (**A**) Percentage of inhibition as a function of time. (**B**) Nyquist diagram obtained in open circuit mode, with 10 mV amplitude and frequency range of 0.1–100,000 Hz for gly/ab-cTnI/AuNP-PAH/LC_{col}/GCE for incubation with 0.3 ng mL^{-1} cTnI in PBS (0.01 mol L^{-1}, pH 7.5) containing 1.0 × 10^{-2} mol L^{-1} of $Fe(CN)_6^{3-/4-}$ to evaluate the effect of incubation time (2 to 60 min). (**C**) Effect of cTnI concentration obtained in the range from 4.18 × 10^{-12} to 1.26 × 10^{-11} mol L^{-1} with incubation time of 10 min as a function of the variation in R_{ct}.
